# Two Clinical Symptoms

**Published:** 1883-05

**Authors:** Clarence Willard Butler

**Affiliations:** Montclair, N. J.


### CLINICAL BUREAU.

### TWO CLINICAL SYMPTOMS.

CLARENCE WILLARD BUTLER, M. D., MONTCLAIR, N. J.

*Read before the N. J. State Homœopathic Medical Society.*

The knowledge which we, as homœopaths, have of the sphere of usefulness of any drug is derived from one or more of four sources:

(*a*) The records of drug provings, or the effects of the drug taken, with a view to ascertain its action, by persons in health.

(*b*) The records of toxicological provings, or the effects of the drug administered, either maliciously or accidentally, in excessive and inordinate doses.

(*c*) The records of sick provings, or the effects of the drug, other than curative, after its administration to persons suffering from disease.

(*d*) The records of curative action, or the disappearance of certain pathological conditions, or symptoms which have never been noticed among those produced by the drug, after its administration when given empirically or as indicated by other and known conditions or symptoms.

That entire and implicit reliance cannot be placed upon any of these sources of information will not be a matter of wonder when we consider the many difficulties which surround the recorders in any or all of these fields of labor. The many modifying circumstances dependent upon unobserved conditions in the provers, upon their habits, their surroundings, the many accidents of every-day life, though in its evenest walks, can be only partially understood, and their influence upon the prover only partially estimated. Then, too, the lack of ability on the part of observers or recorders to express themselves lucidly in recounting their symptoms often leaves their meaning too ambiguous or too vague for practical usefulness; while the unconscious but often undoubted prejudice in the mind of one or the other toward or against certain lines of action (which the utmost care seldom enables them wholly to set aside) tends to exaggeration of certain symptoms at the expense of others equally valuable.

With the records of toxicological provings arise new difficulties. The extreme danger of the involuntary prover, the necessity for prompt action for his relief or for his salvation even, the consequent administration and use of antidotal drugs and measures, the necessary haste in extreme cases, the inability of the sufferer to tell of his sensations intelligibly—all combine to render the records of such cases unsatisfactory. Perhaps their most undoubted value is in presenting pathologico-anatomical changes—a knowledge of which we should never obtain from voluntary provers, for reasons which are sufficiently obvious.

In the case of provings upon the sick, to most of the difficulties already mentioned must be added the almost insurmountable one of determining, among many symptoms of diseased conditions, those which are dependent upon the causes of the original disease, and those which are directly referable to the influence upon the organism of the administered drug. That there is no exaggeration in speaking of these difficulties as "almost insurmountable," that such symptoms and conditions must be accepted with great caution, as the observations of different prescribers upon the persons of the sick under different diseased conditions, are propositions so evident that argument toward their proof is superfluous.

The knowledge which is derived from the curative action of a drug must be received with greater caution than that obtained from any other source. Here we depart from the natural (and better) order of our labors toward the knowledge of drug action—the sick-making power—and study it from the opposite standpoint—its well-making power. The various and shifting aspects of disease, the always active *vis medicatrix naturæ,* the intensified influence of a very natural prejudice on the part of the prescriber in favor of certain medicaments or methods in treatment, combine with many of the obstacles already mentioned to render correct inference extremely doubtful; and I apprehend that more errors have crept into our materia medica from this than from all other sources combined. While so many and such grave difficulties interfere with our ability to determine the exact influence upon diseased conditions of exhibited remedies, too much caution cannot be exercised in admitting to our materia medica symptoms which are entirely clinical. But, so long as many of our drugs lack thorough proving, and the usefulness of such knowledge cannot be questioned, clinical symptoms, which have been repeatedly confirmed by different and conscientious observers, must be admitted as genuine; since doubt of their having been cured by the remedy will, under such proof, yield to certainty, and "under the law," they are those which the drug was capable of producing.

It is for the purpose of calling your attention to two clinical symptoms which I have come to depend upon in my own practice, and to ask you to unite your experience and observation with mine in confirming or disproving them as they may deserve, that I have written this paper. The first is the symptom, "Thirst for *hot* drinks," noticed as cured by *Eupatorium purpureum.*

In THE HOMŒOPATHIC PHYSICIAN for August, 1881, I recorded two cases of intermittent fever, in which this symptom was very marked—one of the later ones to appear in the disease, and one of the first to disappear after the administration of the drug; and, in both cases, this remedy alone was sufficient to the complete cure of the ague. To these I desire to add the following case:

F. W., aged thirty-two, of dark complexion, slight build, and short stature, on the afternoon of March 3d, 1882, became chilled while perspiring from horseback exercise. During the evening and night he suffered from bone pains, heaviness, and languor, and general chilliness. I saw him at 9 A. M., March 4th (the next morning), and found the following condition: A severe, general coldness, with occasional shudderings and chattering of the teeth, bone pains mostly in the arms, legs, and chest. He craves hot water, which he takes in moderate quantities because it relieves his general condition, but especially his coldness. I gave him *Eupat. pur.* 200th, in water, to take a dose every ten minutes until better, and then stop the medicine until I should see him in the evening. At 8 P. M. I received the following report: He took but two doses of the *Eupat. pur.* at intervals of ten minutes, when his chill had nearly disappeared, and he began to feel generally better. Through the afternoon he took two or three doses of the medicine, because he "thought he ought to take something." His bone pains are nearly gone, skin natural to touch, his thirst has entirely disappeared, and he feels quite comfortable. The next morning he presented himself with a severe coryza, which yielded to appropriate medication.

Although I am well aware that the length or continuance of a chill, the direct result of a "fresh cold," cannot be prognosticated; still, its prompt disappearance, with the rapid relief of the attendant symptoms after the administration of the remedy, lead me to present the case as confirmatory of the previous observations of its curative power, especially, of course, with reference to the symptom already cited—the "thirst for hot drinks," and which, in this, as in the two cases referred to, it relieved with the attendant sufferings.

I have repeatedly confirmed the symptom, "Relief from stimulants," as a condition of the pains of *Gelsemium*. The two cases following will illustrate its usefulness:

October 10th, 1880.—Mrs. J. C. B., a tall, strongly built woman, 62 years of age, of sallow complexion and phlegmatic temperament, consulted me for a severe throbbing headache, felt all over the head, but worse in the right side, and especially in the right temple and right eye. It has come on every day for over a week, in the morning between 10 and 11 o'clock, and grows gradually worse till toward midnight, when she gets some sleep, which relieves the pain. It is aggravated by noise, motion, and the recumbent posture. Light does not affect it. I gave her *Belladonna.* The next day (Oct. 11th) she reports, at 9 A. M., that there was no change of any kind noticeable in yesterday's headache, and adds, "I was obliged to take brandy again yesterday." Inquiry shows that the stimulant greatly relieves the pain, not entirely removing it, but enabling her to attend to her household duties, which she cannot do without it. *Gelsemium*, in the 200th potency, given in water, a dose hourly, so modified the severity of the pain that day that she did not resort to the stimulant. The next day there was a slight return of the headache at the usual time, after which she was entirely free from it.

H. W., aged 56, merchant, dark complexion, nervous temperament, consulted me in September, 1879, for the following condition of fifteen or eighteen years' standing: He has every day more or less pain in his chest, which he describes as hard and continuous, coming on gradually, sometimes in one location and sometimes in another, but usually in the præcordial region, lasting for periods of time ranging from a few minutes to two or three days. When present in its severer forms, it wholly incapacitates him for work of any kind, and sometimes results in the following sufficiently alarming symptoms: He loses consciousness, his hands and feet become icy-cold and are spasmodically rigid, the face and trunk are bathed in cold perspiration, respiration is slow, labored, and irregular, and the pulse is hardly perceptible. This condition is usually the culmination of continued and severe pain in the region of the heart, but it has come on quite suddenly without prolonged pain as a precursor. Severe præcordial pain, however, always accompanies this condition. These pains, in some degree, are brought on by continued mental labor, by anxiety—as business or other cares—and "worry," and by unusual physical exertion. Walking will bring it on, if for an unusual distance, and especially walking up-hill. It is almost always relieved or held in check by stimulants of some kind, and for this purpose he has used quinine and alcoholic stimulants in various forms. Brandy in severe attacks and ale in milder ones have afforded the promptest and most satisfactory relief. His habits are regular and good, with the exception of the use of tobacco, which he chews in excessive quantities, and has from his youth. He smokes exceptionally and moderately. He is in the habit of using purgatives of various kinds, and has taken many drugs for the relief of his pain, but without beneficial results. Physical examination reveals no organic heart disease. I diagnosed his condition as one of lowered nervous tone, dependent upon the depressing influence of the tobacco upon his nervous system—in short, as one of chronic tobacco poisoning. Of course this condition was aggravated by his drugging especially by the purgative. The acute pains were undoubtedly neuralgic. My advice was that he stop his tobacco or, at least, moderate its use; but this he protested he was unable to do, and he did not. The use of purgatives was discontinued, of course. I gave him *Gelsemium* 3d, to be used in water should a severe paroxysm of pain come on; and put him upon *Gelsemium*$^{m}$ (Fincke), a dose each night. Under this treatment his improvement was marked and rapid. The first month, during which time he was working unusually hard, he was called upon by severe pain to use the lower potency of the *Gelsemium* three times. He then reported that he felt the pains very seldom—never, unless he became tired or worried; and I directed that he should discontinue regular medication, and only take it when forced to do so by a return of his old enemy. Until this time he has had no severe paroxysm; and although he has been obliged at times to resort to the *Gelsemium* (which he now uses in the 200th potency), it has relieved him promptly. Probably we can expect no more than this while he uses tobacco in his present inordinate manner.

